# Professional perspectives on service user and carer involvement in mental health care planning: A qualitative study

**DOI:** 10.1016/j.ijnurstu.2015.07.008

**Published:** 2015-12

**Authors:** Penny Bee, Helen Brooks, Claire Fraser, Karina Lovell

**Affiliations:** EQUIP, School of Nursing, Midwifery and Social Work, University of Manchester, Oxford Road, Manchester M13 9PL, United Kingdom

**Keywords:** Care planning, Mental health, User involvement, Service delivery, Qualitative research

## Abstract

**Background:**

Involving users/carers in mental health care-planning is central to international policy initiatives yet users frequently report feeling excluded from the care planning process. Rigorous explorations of mental health professionals’ experiences of care planning are lacking, limiting our understanding of this important translational gap.

**Objectives:**

To explore professional perceptions of delivering collaborative mental health care-planning and involving service users and carers in their care.

**Design:**

Qualitative interviews and focus groups with data combined and subjected to framework analysis.

**Setting:**

UK secondary care mental health services.

**Participants:**

51 multi-disciplinary professionals involved in care planning and recruited via study advertisements.

**Results:**

Emergent themes identified care-planning as a meaningful platform for user/carer involvement but revealed philosophical tensions between user involvement and professional accountability. Professionals emphasised their individual, relational skills as a core facilitator of involvement, highlighting some important deficiencies in conventional staff training programmes.

**Conclusions:**

Although internationally accepted on philosophical grounds, user-involved care-planning is poorly defined and lacks effective implementation support. Its full realisation demands greater recognition of both the historical and contemporary contexts in which statutory mental healthcare occurs.

**What is already known about the topic?**

• Involving service users and carers in mental health care planning is central to international health policy and practice. Despite these long standing initiatives, the majority of users and carers still feel marginalised during the care planning process.
• Service users are motivated to collaborate in care planning but substantial barriers are created through poor information exchange and insufficient opportunities for participatory decision making.
• The perspectives of the professionals who are tasked with providing the majority of care to mental health service users have traditionally been under-represented.

**What the paper adds**

• Care planning is a meaningful platform with which to involve service users and carers in mental health care but this involvement is poorly defined and lacks effective implementation support.
• Full realisation demands greater recognition of the historical and contemporary contexts in which statutory mental healthcare occurs.
• Professionals identify on-going training requirements particularly in relation to user centred communication and relational skills.

## 1 Introduction

Involving service users and carers in mental health care planning and promoting shared decision making are central tenets of contemporary mental health policy (Commonwealth of Australia, 2009, Department of Health, 1999, Department of Health, 2000, Department of Health, 2008, HM Government, 2011, World Health Organisation, 2012). Over the last thirty years, outmoded concepts of paternalism, in which clinicians’ beliefs and attitudes have been allowed to dominate treatment decisions, have progressively been eroded, first by a growing consumer movement and latterly by morally and philosophically accepted concepts of therapeutic partnerships, relational equipoise and service user expertise. A dominant choice agenda is now a central cross-cutting principle of the World Health Organisation's Mental Health Action Plan (2012).

Consumerism infused the health policy of many countries in the 1980s as part of a market ideology that promoted individual patient choice and acknowledged the importance of healthcare satisfaction (Tait and Lester, 2005). In the United Kingdom, such concepts were gradually developed and expanded to include an acknowledgement of patients as experts in their own illness and thus an element of reciprocal responsibility in care planning and treatment decisions (Hickey and Kipping, 2002). Today, user and carer involvement is an established policy mandate, most recently consolidated by a new personalisation agenda for adult social care across England and Wales (Department of Health, 2008, Healthcare Commission, 2008b, HM Government, 2011, Secretary of State for Health, 2012). Similar developments have occurred overseas, with international research and policy imperatives upholding the importance of participatory mental healthcare and its perceived role in improving the culture and responsiveness of services and the quality of care that users receive (Commonwealth of Australia, 2009, Daremo and Haglund, 2008, Goodwin and Happell, 2008).

The central and unfailing premise of modern health policy is that users and carers are major stakeholders in service delivery and, as such, must be regarded as participants rather than simply recipients of mental health care. Small scale studies suggest that involving service users and carers in the planning and delivery of care can have positive effects on service and individual outcomes (Simpson and House, 2002, Thornicroft and Tansella, 2005); reducing rates of enforced admission and treatment for people with severe mental illness (Henderson et al., 2004), increasing user esteem, and empowering individuals to regain control over their own recovery and care (Henderson et al., 2009). Yet, despite the philosophical and empirical support for user involvement, substantial evidence suggests that its translation into practice has not been easy to achieve.

At the beginning of the new millennium, Peck et al. (2002) proposed a framework for user involvement in mental health services, with user participation operating at four main levels. These levels pertained to (i) interactions between service users (self-help), (ii) interactions between users and health professionals (individualised care planning), (iii) local service management opportunities and (iv) service planning. Although the application of this framework in a UK setting identified a growing diversity of involvement initiatives, the majority of activities remained at a tokenistic level, with service users framed predominantly as subjects of consultation rather than agents in control (Tait and Lester, 2005). The strength and consistency of the views garnered at the time led some commentators to assert that there existed ‘conflicting evidence as to the existence of [involvement] philosophies in the reality of mental health nursing practice’ (Anthony and Crawford, 2000).

A recent systematic review of the international literature, specifically focused on user involvement in mental health care planning, reveals comparable findings (Bee et al., in press). Although substantial evidence suggests that users are sufficiently motivated to collaborate in care-planning, substantial barriers continue to be created through poor information exchange and insufficient opportunities for participatory decision making. National and international research literature is consistent in indicating that the majority of users and carers feel marginalised during the care planning process (Jakobsen and Severinsson, 2006, Mental Health Council of Australia, 2000), and that this lack of involvement occurs in both inpatient and community settings (Care Quality Commission, 2009, Healthcare Commission, 2008a). At best therefore, policy imperatives remain inconsistently implemented, and at worst are challenged or diluted by more ritualised practice.

Implementation theory suggests a number of possible explanations for the sustained translational gap. These include individual or communal appraisals of the concept and worth of user involvement, the quality of the relationships that exist between stakeholders, the organisational environments in which these relationships occur, and the autonomy and capacity of the implicated agents in facilitating change. Early small scale studies emphasised the potentially prohibitive roles of organisational influences (Anthony and Crawford, 2000); finite resources (Bowl, 1996) and professional resistance to user involvement (Crawford et al., 2003), with observed differences between mental health professionals’ outward support for collaborative engagement and service users’ perceptions of their frontline behaviour (Campbell, 2001).

Less clear are the reasons why such discrepancies exist and to what extent they continue to impact on contemporary mental healthcare practice. Potential reasons include conceptual differences in the interpretation and meaning of involvement between service users and professionals (Bee et al., in press), variability in the extent to which these two parties adopt more scientific or social ways of thinking and working (Summers, 2003), and/or professional resistance to sharing or transferring power. Distinguishing features of mental health services are acknowledged to include a long standing history founded on aspects of containment and compulsion and an entrenched stigmatisation of service users (Munro et al., 2006). Nonetheless, initial concerns that effective involvement might be barred by illness severity (McDermott, 1998), or undermined by treatment refusal (Atkinson and Gilmour, 2004), have rarely been realised in practice (Papageorgiou et al., 2004).

The ongoing sense that current models of service user and carer involvement continue to be less effective than first envisaged gives rise to an increasingly urgent need to elucidate the reasons why. Although contemporary health services are progressively utilising a wider range of user involvement strategies (Crawford et al., 2003, Tait and Lester, 2005), professional opinion is still alleged to dominate the majority of nursing practice (Goss et al., 2008). Pertinently, mental health nurses, psychiatrists and allied health and social care workers continue to provide the majority of care for mental health service users, yet in-depth explorations of their perceptions of user and carer involved care planning are sparse. Rigorous, qualitative studies of care planning implementation are limited and thus so is our understanding of the individual and service perspectives sustaining this important translational gap.

### 1.1 Aim

To explore contemporary mental health professionals’ perceptions and experiences of delivering mental health care planning and involving service users and carers in decisions about their care.

## 2 Method

### 2.1 Study design

Given the exploratory nature of our research study, we utilised a qualitative approach incorporating two different data collection methods (focus groups and individual interviews).

### 2.2 Participants

Participants were recruited from two large NHS Trusts in North West (Focus groups and interviews) and Central England (Interviews only). Recruitment strategies comprised advertising on Trust intranets, newsletters and press releases and displaying posters within Trust premises and the use of Trust based co-applicants to ‘champion’ the study. All mental health professionals working with service users within the Trusts’ geographical areas were eligible to participate. Purposive sampling strategies were employed to ensure adequate representation across gender and occupational roles.

Potential participants contacted a named member of the research team in order to ascertain eligibility, discuss availability, be allocated to a focus group or arrange a suitable interview date. All interested parties were sent a study information sheet in advance of attendance and were routinely contacted by a researcher before data collection to be given the opportunity to ask questions. Before the start of each focus group or interview, participants were given a second opportunity to ask questions prior to giving written consent.

### 2.3 Data gathering

Self-reported demographic information (gender, ethnicity and occupational role) was collected from each participant consenting to take part. Participants took place in either a focus group or interview and not both.

Four focus groups were carried out with mental health professionals (with a total of 23 participants) on Trust and University premises between June and September 2013. Focus groups lasted between 55 and 88 min (mean of 68.5 min). Each group was managed by a team of 2–3 researchers covering the roles of lead facilitator, co-facilitator and participant welfare support. Wherever possible, the team included a trained service user/carer researcher and both participants and service user/carer researchers were given an opportunity to opt out of a particular group if they had a prior therapeutic relationship. Focus group schedules covered current perspectives on user involvement in care planning, the processes and outcomes of care planning, prior experiences of service user/carer involvement and potential training requirements. Groups were made up of a range of different professionals incorporating a range of positions. Focus group leaders discussed the importance of valuing and respecting the opinions of other and confidentiality and made sure each participant felt comfortable taking part and voicing their opinions prior to the focus group starting.

Twenty-eight individual interviews were completed between June and October 2013. These were conducted face to face on Trust and University premises and lasted between 36 and 95 min (mean 63.0 min). Semi-structured interview schedules explored individual appraisals of care planning and user/care involvement, current organisational processes and systems related to care planning and the potential success of otherwise of future training initiatives. All focus groups and interviews were digitally audio-recorded, transcribed verbatim and anonymised prior to data analysis.

In line with ethical guidelines, a distress protocol was followed if any participant showed signs of distress; this was not required during any focus group. The distress protocol was activated for two professionals participating in individual interviews; in one case, audio recording was stopped and subsequently resumed in line with the participant's wishes. In the other, distress occurred after the interview had finished. Both participants indicated that they wished to remain in the study.

### 2.4 Data analysis

Recorded interviews and focus groups were transcribed by an experienced, independent transcription company. Transcriptions were anonymised upon receipt and allocated to a member of the research team (HB, CF or PB) for preliminary analysis. Analysis followed a qualitative framework approach (Ritchie and Spencer, 1994), a popular way of analysing primary qualitative data pertaining to health care practices with policy relevance (Dixon-Woods, 2011). Framework analysis permits both deductive and inductive coding, enabling potentially important themes or concepts which have been identified a priori *to be* combined with additional themes emerging de novo.

Framework analysis incorporates five phases (Furber, 2010); familiarisation, developing a theoretical framework, indexing, charting and synthesis. To summarise this approach, authors read and reread their transcripts to familiarise themselves with the data before undertaking initial transcript coding. Once the first 10 transcripts had been coded, authors met to discuss new emergent codes and to develop a shared theoretical framework from the codes. This was then applied to the remaining transcripts and team meetings were held to discuss any further revisions to the framework that resulted from this analysis (see Appendix 1 for a copy of the original framework).

An excel spread sheet was developed which incorporated preliminary framework themes as column headings and the demographic information related to participants who provided data under each theme. As the constant comparison of new data occurred and the authors’ understandings of the themes under consideration developed, the framework was amended and re-shaped to enable the introduction of new codes and/or the deletion of redundant, similar or otherwise compromised codes. In this way, a final framework was achieved that was considered representative of the entire dataset that was collected.

### 2.5 Reducing bias

Regular meetings between the research team ensured that the emerging codes remained grounded in the original data. Emerging codes were presented to a wider project panel at study synthesis day in November 2013. Members of this panel which included service users and carers, who had not participated in data coding or analysis prior to this point, were asked to comment on the grounding of the data and any perceived ambiguities or gaps in the emergent framework.

## 3 Results

**Fig. 1:**
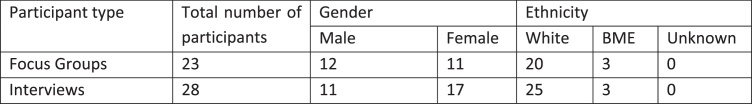
Demographic information relating to study participants.

Demographic information relating to the participants who took part in the interviews and focus groups can be found in Fig. 1. In total, 9 participants (18%) were in management roles, the remainder (*n* = 42, 82%) working directly with service users/carers. Host services included crisis teams; community mental health teams; later life/dementia teams; inpatient services; psychiatry; dual diagnosis and specialist drug/alcohol services; recovery services; mental health advocacy and occupational therapy.

Our analysis revealed clear similarities and overlap in the perspectives of different mental health professionals working in different roles and settings. Thus, although we report professional characteristics alongside anonymised quotes for clarity, we do not present differences in the views of different healthcare professionals.

### 3.1 Care planning as a meaningful platform for user and carer involvement

Consensus among study participants was that care planning comprised a core element of the nursing ‘toolkit’, with multiple roles to play in needs assessments, care delivery, inter-professional communication and audit. The strength and consistency of these views was striking and reflected a strong bias towards care planning conceived as a service-led event.

“*So I think they’re [care plans] useful as, kind of, a framework and as a prompt, but I guess it's making sure that you revisit that and you come back to that.*”

**101 MALE*****,*****COMMUNITY PSYCHIATRIC NURSE**

Simultaneously, many professionals advocated service user and carer involvement in mental health services. The vast majority of respondents were consistent in attributing worth to user involvement, and in perceiving user involved care planning to confer unique and multiple benefits. Explicit recognition of differences in the life history and lived experience of service users and professionals provided the logical justification for this stance, with user and carer involvement being perceived to contribute otherwise unavailable information to the decision making process. Other benefits of user involved care planning extended beyond this immediate care context, most commonly including notions of improved stakeholder relationships, reduced power differentials, improved care quality and reduced long term demand on health and social care services.

“*Because no matter how hard you try and put yourself in somebody's shoes, you’re not them, so it would just be really great for somebody to go and kind of…I’m sure there's loads of stuff that, you know, you just never think of, in the position of the nurse, you know, you try and be, you know, empathic and of course that's your job, but, you know, I think there's definitely loads of things that I would just never have even thought of.*”

**102 FEMALE, STUDENT NURSE**

“*Because if the person is included, it's for them and they’ll participate fully and they will, you know, they will…it will help them…their wellbeing… .instead of just being told, you have to do something and I can’t do it, because I think the stressful thing is, when you’re getting told to do something and your culture doesn’t allow you to do it, or the timing isn’t right…*”

**103 FEMALE, BME RECOVERY WORKER**

### 3.2 Philosophical tensions in user-involved care planning

Despite widespread support for the ideology of service user involvement, user involved care planning was deemed difficult to realise in practice. Marked tensions were identified between contemporary care philosophies advocating patient empowerment and long standing socio-medical constructs of mental health services founded on aspects of safety and containment. Most participants perceived deeply engrained nursing behaviours, sustained through top-down, risk averse cultures to challenge the integrity of user involvement, prohibiting meaningful engagement and conflicting directly with service user concepts of hope and recovery. Risk management was identified as one of the fundamental roles of mental health care planning, and as such featured as a core discussion topic for all participants.

“*I think some professions would find that quite scary and quite daunting, and actually, you know, thinking about how that might reflect on them as practitioners. And if anything happened, and they were up in coroners court or, you know, being disciplined, actually how would it reflect for them to not have fed in as a professional with all this knowledge. But, I think it would actually be a really positive thing, people would be working towards what they want to do and you would get better results for the person really.*”

**104 FEMALE, OCCUPATIONAL THERAPIST**

Genuine user involvement, whilst desirable in principle, was deemed to necessitate a marked shift in organisational ethos. This shift was believed to be necessary to move employers and employees away from traditional models of accountability, enabling professionals to promote user independence and self-management without fear of litigation or reprisal. The term ‘positive risk taking’ was used in this context to represent professional readiness to respect and respond to service users own recovery goals or preferences for care. Such opportunities were perceived to be under-realised in practice, exacerbating existing power differentials between service users and professionals and sanctioning professionals to have the ‘final say’.

“*But it's all about all those areas of people's lives that are seen…so you can have a fulfilling life……so it isn’t just risk, meds which is normally what tends to happen.*”

**104 FEMALE, OCCUPATIONAL THERAPIST**

### 3.3 Individual and contextual differences in service user involvement

Exacerbating a philosophical tension was the recognition that secondary mental health care teams were increasingly being asked to accommodate a multiplicity of diagnoses, illness severities and user experience. As a consequence, respondents described how operational constructs of optimal user involvement had become increasingly difficult to define, with potential variation in staff and user preferences and/or user capacity demanding a high level of fluidity in care planning implementation and design. Clear differences between care environments as well as individual fluctuations in illness severities led to specific difficulties in crisis settings, where lack of insight and fast-changing needs were often felt to preclude traditional models of service user involvement.

“*In crisis team, we don’t give the patients a written care plan, I’ve, you know, I’ve been away from the crisis team several months and now come back, and I see, I’m guessing about only half of the patients have actually got a care plan. … But, it's fairly fast changing, you can imagine they would always, by the time they’re printed and taken round the plan would have changed.*”

**FOCUS GROUP PARTICIPANT A, ROLE NOT IDENTIFIED**

Insight was deemed a key factor limiting joint deliberation. For some this was presented as a clear rationale for maintaining close relationships with service users, co-developing advance directives and maximising user involvement during periods when individuals were well. For the majority however, lack of insight was viewed as a direct contradiction to the philosophy of user involved care planning. Consistent with a bias towards paternalistic, medical models of care, participants discussed how it was almost impossible to undertake user-involved care planning in the absence of a psychiatric diagnosis. Additional challenges were experienced for specific diagnoses (e.g. personality disorder) and for mental health service users with substance misuse co-morbidities. These individuals more than any other were perceived to present staff with excessively chaotic lifestyles, competing behaviours and care agendas which in turn limited their own confidence and ability to sustain open and honest care planning dialogue.

“*I would guess, I mean sometimes, yeah, sometimes the person maybe is too poorly to really be involved. Which then is difficult but then I think sometimes…so a care plan gets written without their involvement because they’re too, you know, poorly to have a discussion about it, but then maybe they’ll get a bit better and it will be forgotten that maybe now that they could be involved in a discussion about care.*”

**FOCUS GROUP PARTICIPANT B, ROLE NOT IDENTIFIED**

Although service user desire and capacity for involvement were perceived to vary, there was also recognition that, in the absence of any shared definition of optimal involvement, professionals’ themselves had formulated their own individual models of collaborative care planning. Within such models, scope for misinterpretation was evident, both in terms of their own conceptualisations of service user involvement, and the assumed capacity and readiness of users to be involved.

“…*some people are very offended and angry that you’ve sent them a care plan, particularly if it's got Manchester Mental Health and Social Care printed on the top of it, they don’t like that. Er…there's a lot of stigma still around Mental Health Services, erm…some people would struggle to understand why you’ve sent it to them and would be saying what's this, you know, I don’t understand it, don’t understand what you’ve written. So you might have to explain that, and some people I think just chuck them in the bin*.”

**105 FEMALE, LATER LIFE TEAM**

### 3.4 The feasibility of user involvement in practice

Irrespective of service setting, professional discourse was clear in describing the existence of an ‘organisational shift’ in user involved care planning, created and sustained by ill-conceived notions of the feasibility of user involvement, and a potential mismatch between the rhetoric of service leaders and the organisational culture and workload pressures faced by front line staff. Translational gaps were most likely to occur where staff were overburdened with administrative responsibilities, where the culture, language and terminology of a service were not yet user-centred and where staff were not legitimised in providing holistic support. Acceptance of these issues may require a broader service overhaul.

The greatest barrier reported by health professionals to involving service users and carers in care planning was increasing time and workload pressures. Participants often felt that the sheer volume and complexity of their workload meant they did not have enough time to spend with users and carers, thereby limiting the nature of the relationship that could be established between them and by implication the amount and quality of involvement that could be achieved. Service managers also reported a lack of time to dedicate to supervising the care planning practices of junior staff, with the net effect that teams were left vulnerable to pre-existing paternalistic practices and dominant ritualistic cultures. Distinction was drawn between the flexibility required to achieve meaningful user involvement on the one hand, and the pursuit of an efficient, cost–effective health service on the other. It was felt that this incongruity had ultimately led to the relegation of user involved care planning in favour of the administrative efficiency promoted and prioritised under a target driven culture.

“*There are so many targets within the Trust at the moment with regards to, kind of, paperwork that clinicians have to complete. So I suppose it would, kind of, refocus the meaning behind it, um, because I think sometimes that can be lost.*”

**101 MALE, COMMUNITY PSYCHIATRIC NURSE**

### 3.5 Relational skills as a facilitator of service user involvement

Due to the lack of a defined implementation structure for user involved care planning, mental health professionals were clear that responsibility and motivation for user involvement lay predominantly with front line staff. The qualitative nature of the relationships established between professionals and users was posited as a key determinant of successful user involvement, and one capable of overcoming a lack of wider organisational support. Within this context, staff talked freely about the difficulties they had experienced in engaging some carers by implication involving them in the care planning process. Current care planning training was viewed as ‘ad-hoc’ and infrequently reviewed, with a lack of a standardised training package in pre and post registration courses.

“*It's about getting them all to sing from the same hymn sheet and having that continuity and consistency in your care plan. Because that continuity and that consistency is the thing that's going to get you better. Not someone saying this, someone saying that and you saying this. It's that's continuity and consistency. Do it, put that through in a training package somehow and then it might start to work.*”

**FOCUS GROUP PARTICIPANT C, ROLE NOT IDENTIFIED**

Although a minority of participants rejected the notion that additional training would be beneficial, group consensus suggested fundamental skill deficits in user involvement and a lack of awareness of effective models for engaging service users in care planning discussions. Specific training requirements identified by staff included revisiting fundamental listening and engagement skills and extending this to service users who lacked insight, understanding engagement and involvement from the service user perspective, managing service users’ expectations in the context of limited economic resources, and increasing sensitivity and response to the needs of Black and Minority ethnic groups.

“*The bigger part of it should be about how do you deliver collaborative care planning, shared decision making, language, attitudes, what are some of the tools that are around and available for people to enhance that ability to do shared…how do you give power to the person receiving services? So a lot of that I think the doing should be focused much more on that, on those aspects.*”

**106 FEMALE, PROJECT MANAGER (RECOVERY)**

Central to professional discourse, was the idea that explicit strategies could be learnt to enable staff to confront and overcome difficult issues that commonly occur. These included balancing meaningful carer involvement with other, potentially more engrained, codes of conduct (such as maintaining user confidentiality) and negotiating successful user involvement alongside professional and legal accountabilities. A substantial proportion of participants described the process of user involved care planning as particularly complex, necessitating a change in role that was not intuitively compatible with other responsibilities under statutory mental health law. Training was required to enable staff to challenge service user stigma, reject ritualistic, service-centred practices and mentor additional members of the clinical team.

“*I think that they can use their own…the service users can use their own experience to inform their training, in ways which…which really confront attitudes and values, and stigma around mental ill health, and explode some myths.*”

**107 MALE, DUAL DIAGNOSIS WORKER**

## 4 Discussion

We conducted a qualitative analysis to gain a deeper understanding of professionals’ views of the ideology of user and carer involvement in mental health care planning. Our data has highlighted an important disjuncture between the rhetoric of user involvement upheld by international mental health policy (e.g. Commonwealth of Australia, 2009, HM Government, 2011, World Health Organisation, 2012) and the propensity and capacity of readiness to adopt such practices on the ground. Whilst this observation is not new, the reasons underpinning and fuelling this sustained translation gap are critically important to note.

The international relevance of our work lies in its capacity to address ongoing dissatisfaction with service user involvement and to overcome substantial translational delays. User participation in healthcare is a government mandate in many states, driven by consumer-orientated health agendas in combination with rising democratisation of decision making processes (World Health Organisation, 2012). Yet, its potential to transform services has historically not been realised, with systematic reviews highlighting consistent user and carer requests for greater information provision, more meaningful treatment choices and increased involvement in care consultations (Bee et al., 2008, Bee et al., in press).

Acknowledgement of the entrenched stigmatisation of service users and/or debate regarding nurses’ failings to deliver compassionate care provide two potential rationales for the observed service gaps. However, the current data begins to challenge these popular cultures of blame. Our findings do not preclude the possibility that attitudinal barriers exist, but it is clear that much more can be done at organisational and policy level to enable user involvement to occur.

Consensus amongst the wide range of mental health professionals included in our study was that care planning was a core element of the nursing toolkit, ripe for quality and content enhancement through user and carer involvement. Collaborative participation, although supported at an ideological level, was commonly barred by a potent combination of conceptual and operational delays. Conceptual delays were underpinned by a lack of shared understanding regarding the concept of user involvement and specifically the extent to which it aligned or jarred with supplementary codes of conduct based on professional accountability and risk. Organisational delays included a lack of institutional time and space for user involvement and an absence of clinical guidelines and managerial support through which to define and operationalise optimal involvement strategies.

The routinisation of any new policy initiative is recognised as a complex process, with current models evolving out of diverse disciplines including knowledge transfer and quality improvement. Our data suggests that, whilst not contended on philosophical grounds, the pace of the international user involvement movement has not yet been matched with effective implementation support. Pragmatic omissions, such as those related to the provision of training and practice resources, potentially take on additional significance against a historical backdrop of unequal power relations and the potentially different interests of ‘opposing’ stakeholders (Lewis, 2014). The discourse surrounding ‘service user involvement’ has itself been criticised for defining individuals predominantly in terms of their contact with mental health services, with the implicit acknowledgement that the potentially negative status of psychiatry in society remains unaddressed (Roberts, 2010, Lewis, 2014). Future success may thus depend upon greater recognition of the historical, cultural and contemporary contexts in which the majority of secondary mental health care occurs.

The emergence of service user insight as a key influence on care planning practice is an important finding. Reduced insight has long been accepted as a reason for the adoption of more paternalistic approaches to care and as a potential limiter of participatory decision making (Szmuckler, 1999). Similar to recent studies exploring shared decision making for anti-psychotic medication (Shepherd et al., 2014), our data suggests that health professionals may tend towards concepts of insight as a dichotomous rather than continuous variable. Concepts of insight as either present or absent have already been shown to conflict with users experience of psychosis; with qualitative studies suggesting that autonomy should be respected as far as possible in care consultations (Laugharne et al., 2012).

Any initiative seeking to become a routine part of practice requires stakeholders to create a level of stability, derived from a common understanding of its purpose and advantage. Encouragingly, our study has shown that professionals rarely conceptualise involvement as a static feature of practice but as a potentially beneficial and fluid process reflecting a dialogue that is both contextually and temporally dynamic. This new observation highlights a potentially critical role of boundary clarification and the important work that still needs to be done in developing consensual accountability systems and operational guidelines for practice.

Our qualitative analysis has helped to elucidate some of the ways in which the mental health community is still engaged in the reworking of conventional, paternalistic practice, alongside the projection of new roles and resourcing into the future. The success of the intentional user involvement agenda is likely to rely heavily upon the success of these efforts and concomitantly, local and national investment in staff development. Our study suggests that the skills and confidence of front line staff required to adopt and practice user involved care planning are likely to vary substantially across and within service settings.

By failing to provide adequate systems transformation, the rhetoric of user and carer involvement has historically fallen to individuals, whether users and providers, capable of functioning as localised ‘involvement champions.’ Professional narratives reflect a sense of inconsistent or constrained implementation, determined largely by the self-perceived confidence and competencies. By identifying such individuals, and shedding light on a potential mismatch between professional readiness and capacity for implementation, our data suggest that the ideology of user involved care planning continues to hold promise. Encouragingly, its enactment through the provision of appropriate and specialised training programmes may be a fruitful and potentially more immediate way of achieving meaningful participatory practice.

### 4.1 Strengths and limitations

A particular strength of this study is that we conducted focus groups and in-depth interviews with a broad range of mental health professionals. By undertaking these within a semi-structured format, we allowed participants to raise issues that were important to them and which may not have arisen during a quantitative, questionnaire based study. We included professionals from a number of different clinical areas who had a broad range of different experiences of health settings. The data presented in this paper represent necessarily partial views (e.g. only mental health professionals) which were considered important and under-represented in previous literature. The views of service users, carers and other potential stakeholders are presented elsewhere.

We did not undertake ethnographic observation data on care planning interactions, and as such our analysis must remain exploratory. In addition, the views of professionals from only two Trusts were included. Our use of both deductive and inductive approaches to analysis enabled the study to go further than the dominant discourses that frequent mental health policy and explore the individual, organisational and socio-medical influences on involving service users and carers in care planning.

The current study adds support to a wider programme of NIHR-funded research currently being undertaken by the research team (NIHR PGfAR RPDF-1209-10020: Enhancing the Quality of User Involved Care Planning in Mental Health Services). This research is currently trialling the effectiveness of a training programme for mental health professionals to enhance user and carer involvement in care planning. The data presented here have informed the development and content of the training programme. A nested process evaluation running alongside the trial will explore additional issues relating to implementation.  *Conflict of interest*: None.  *Funding*: This paper summarises independent research funded by the UK's National Institute for Health Research (NIHR) under its Programme Grants for Applied Research Programme (Grant Reference Number RP-PG-1210-12007). The views expressed are those of the author(s) and not necessarily those of the NHS, the NIHR or the Department of Health.  *Ethical approval*: Ethical approvals were obtained from NRES Committee North West – Greater Manchester North and Dyfed Powys Research Ethics Committee (ref: 13/NW/0047 and ref: 13/WA/0074).

## Appendix 1 Thematic framework

1. Training
  (1a) Need for training
    • Individual need (staff skills)
    • Organisational need (shared model)
  (1b) Content
    • Engagement and listening skills
      ∘ Importance of engagement–why it matters
      ∘ Practical skill training–how to engage
      ∘ Understanding from service user (SU) perspective
      ∘ Understanding power relationships
      ∘ Involving service users who lack insight
    • Enabling involvement in the context of risk management
    • Enabling involvement in crisis settings
    • Recording and responding
      ∘ Writing style
      ∘ Organisational context/barriers
        - Training must be realistic to current climate (restricted resources)
        - Managing service user goals/expectations in context of limited resources
    • Individual context/barriers
      ∘ Staff mindset (enabling not managing)
      ∘ Being open to change
    • Overcoming difficult issues
      ∘ confidentiality and info sharing with carers
      ∘ enabling understanding of prof code of conduct/standards
      ∘ developing and implementing advanced directives
    • Working holistically with the service user
  (1c) Delivery
    • Graduated format (start with basic skills)
    • Needs real (in person) service users and carers–for impact/genuine engagement
    • Practical Skills
      ∘ Role play
    • Status/Recognition–training must have this
      ∘ Mandatory
      ∘ CPD/accredited
      ∘ Trained trainers
    • Setting
      ∘ Organisation-based delivery–specific to setting
      ∘ Delivered to whole teams to ensure ‘team buy in’
      ∘ Multi-disciplinary delivery
    • Standardisation
      ∘ Consistency across teams/organisations
      ∘ Early career/pre-registration–consistency starts with early training
    • Continued learning
      ∘ DVD to take away
      ∘ Reflective practice
    • Who should attend/recipients
      ∘ All team including manager
      ∘ GPs
  (1d) User/carer involvement in training
    • Benefits
      ∘ Higher impact
      ∘ Higher quality
      ∘ Increased focus/engagement and receptiveness
    • Barriers/difficulties
      ∘ Selection of trainers
      ∘ Ensuring service users and carers are well supported in role
      ∘ Ensuring involvement does not appear tokenistic
      ∘ Staff perceptions
2. User/carer involvement
  (2a) Experiences of UCI
    • Engagement difficulties
      ∘ Cultural/language
      ∘ Terminology (medical)/Written from professional perspective
      ∘ Setting (crisis – pace of change)
    • Extent of involvement
      ∘ Variable experiences
      ∘ Organisational ‘Shift’ – Management versus frontline inconsistencies
      ∘ Understanding/defining user involvement
      ∘ Service user desire for involvement
  (2b) Barriers
    • Organisational
      ∘ Workload pressures/target/culture
      ∘ Litigation culture/risk aversion
      ∘ Economic climate/service/resource cuts
      ∘ Absence of involvement culture
      ∘ Medical model culture
      ∘ Paternalism/professional self concept
    • Diagnosis
      ∘ Lack of or preliminary
    • Understanding
      ∘ Understanding of system/processes
      ∘ Literacy
      ∘ Cultural/language barriers
    • Accessibility
      ∘ Timing of meetings
      ∘ Failure to communicate meeting arrangements
    • Relational barriers
      ∘ Power to detain
      ∘ Ownership
    • Measuring impact
      ∘ Lack of quality outcome measures
  (2c) Facilitators
    • Individual approach/genuine engagement
      ∘ Fluid/flexible approach
    • Holistic approach – seeing the whole person/context
      ∘ Acknowledging and acceptance of risk
        - Negotiating different views
    • Service user ownership of care planning
    • Service user led approach – professional as facilitator
      ∘ Risk negotiation
    • Role of setting
    • Advocate support
    • Strategic buy in (organisational culture)
    • Real-time consultation and co-construction
3. Experiences of Care Planning
  (3a) Purpose of Care Planning – What do staff think it is for?
    • Needs assessment
    • Communication tool
      ∘ Within teams
      ∘ Across teams
      ∘ With service users/carers
    • Audit
  (3b) Mixed Experiences of care planning
    • Context/setting dependant
      ∘ Inpatient settings
      ∘ Community settings
    • Contradictory accounts (frontline staff versus managers)
    • Staff individual differences
      ∘ Individual values/motivation/patience
      ∘ Professional background (matching to service user need)
      ∘ Inter-professional alignment
    • Desire for engagement
  (3c) Positive experiences (‘Good’ CP)
    • System
    • Content
      ∘ ‘Flavour’ of service user/temporal perspective/wider context
      ∘ Strong assessment
      ∘ Specific action plan
      ∘ First person narrative
      ∘ Whole person approach
      ∘ Meaningful risk management narrative
    • Process
      ∘ Facilitates engagement with service users/carers
      ∘ Supports consistent approach
      ∘ Supports multi-disciplinary working
      ∘ Supports patient understanding of care
      ∘ Role of care co-ordinator
    • Staff approach
      ∘ Listening rather than ‘assessing’
      ∘ Not a ‘slave’ to care planning template
      ∘ Works holistically and in a patient-centred way
    • Organisational
      ∘ Good support from organisation
  (3d) Negative experiences (‘Bad’ CP)
    • Process
      ∘ Lack of medic involvement
      ∘ Lack of co-ordinated teamwork/fragmentation
      ∘ Inconsistent data sharing
      ∘ Senior manager ‘distance’ from frontline staff
      ∘ Failure to involve service users and carers
      ∘ Relies on understanding of the system
      ∘ Excludes high risk service users
      ∘ Excludes difficult to engage service users (issue of insight)
      ∘ Over reliance on medical voice
    • System
      ∘ IT system (encourages sectional/cut and paste approach)
      ∘ Structure of system
    • Organisational barriers
      ∘ Target culture
      ∘ Time/workload pressures
      ∘ Economic climate/limited support resources to refer on to
      ∘ Lack of supervision
      ∘ Statutory/legislative impact
    • Content
      ∘ Rigidity
      ∘ Tick box approach
      ∘ Lacks holistic/whole person approach/narrative
      ∘ Restricted content/lacks detail–excludes physical health; cultural, religious, community, sexuality needs
      ∘ Problem focussed (rather than solution focussed)
      ∘ Too much focus on risk management/crisis
      ∘ Professional dumping ground
    • Wider factors
      ∘ Societal response to mental health
      ∘ Negative input from some carers
  (3e) What should care plans look like?
    • Clear expectations (patient's charter)
    • Individual patient-centred/whole-person approach/model
    • Context/history
    • Therapeutic journey (what's worked/not worked)
    • Summary of assessments/decisions made
    • Key messages
    • Meaningful needs-based action plan
    • Time/person sensitive
    • Meaningful risk assessment/management
    • Meaningful user involvement
    • Adequately resourced
    • Outcome domains
    • Acknowledging divergent views
    • Advanced directives
